# Triacetyl Resveratrol Inhibits PEDV by Inducing the Early Apoptosis In Vitro

**DOI:** 10.3390/ijms232314499

**Published:** 2022-11-22

**Authors:** Xue Wang, Yi Liu, Kaiyuan Li, Ming Yang, Qingtao Wang, Zhihui Hao

**Affiliations:** 1 College of Veterinary Medicine, China Agricultural University, Beijing 100193, China; 2 College of Veterinary Medicine, Xinjiang Agricultural University, Urumqi 830052, China

**Keywords:** porcine epidemic diarrhea virus, triacetyl resveratrol, apoptosis, caspase pathway

## Abstract

PEDV represents an ancient Coronavirus still causing huge economic losses to the porcine breeding industry. Resveratrol has excellent antiviral effects. Triacetyl resveratrol (TCRV), a novel natural derivative of resveratrol, has been recently discovered, and its pharmacological effects need to be explored further. This paper aims to explore the relationship between PEDV and TCRV, which offers a novel strategy in the research of antivirals. In our study, Vero cells and IPEC-J2 cells were used as an in vitro model. First, we proved that TCRV had an obvious anti-PEDV effect and a strong inhibitory effect at different time points. Then, we explored the mechanism of inhibition of PEDV infection by TCRV. Our results showed that TCRV could induce the early apoptosis of PEDV-infected cells, in contrast to PEDV-induced apoptosis. Moreover, we observed that TCRV could promote the expression and activation of apoptosis-related proteins and release mitochondrial cytochrome C into cytoplasm. Based on these results, we hypothesized that TCRV induced the early apoptosis of PEDV-infected cells and inhibited PEDV infection by activating the mitochondria-related caspase pathway. Furthermore, we used the inhibitors Z-DEVD-FMK and Pifithrin-α (PFT-α) to support our hypothesis. In conclusion, the TCRV-activated caspase pathway triggered early apoptosis of PEDV-infected cells, thereby inhibiting PEDV infections.

## 1. Introduction

In the context of COVID-19, phytochemical formulations from traditional Chinese medicine (TCM) are now being actively employed as an adjunct strategy to antiviral drugs [1,2,3]. Resveratrol (RSV) was considered as a central component of TCM [3]. Resveratrol has a broad range of antiviral effects on various human and animal viruses [4,5,6]. Triacetyl resveratrol (TCRV) is a derivative of resveratrol that exists in natural plants and has better pharmacological activity than RSV [3,7]. However, there is still a lack of studies on TCRV, and it is necessary to study the effects of TCRV and explore their potential pharmacological value.

Porcine Epidemic Diarrhea Virus (PEDV) is the swine coronavirus (CoV) belonging to alpha Coronaviridae [8]. The incidence of PEDV is high and is still spreading. By the end of 2010, PEDV emerged in China [9,10]. In 2013, an outbreak occurred in pigs of all ages in the United States, with the incidence among suckling piglets being as high as 90% [11,12]. Subsequently, PEDV was prevalent in Canada, Mexico, and other counties [13,14,15,16]. Existing studies have reported that PEDV can exist in transported feeds for a long time, which increases the possibility of its transmission [17]. PEDV causes devastating economic losses to the porcine industry globally. Presently, PEDV has become an important disease that has endangered the global pig industry, and the trend has not yet decreased [18,19]. There is a lack of effective therapeutics to inhibit infection by and spread of PEDV, except for a few vaccines [20,21]. Therefore, finding efficient medicines to control PEDV infection is of great importance.

PEDV infection induces caspase-mediated apoptosis [22]. However, the activation of apoptotic pathways contributes to viral replication at infection sites and to mature virion spread at the cellular and multiple system levels [8,23]. There exists a bidirectional relationship between viral infection and apoptosis [24]. The oligomerization of the Bcl2-associated X protein (Bax) results in the pore formation within mitochondrial membranes as well as cytochrome C release in cytoplasms [25]. This is an important process in apoptosis. Cytochrome C (Cyt C) binds to the apoptotic peptidase activator 1 (Apaf1) and pro-caspase-9 to form “apoptotic bodies”, thus activating caspase-9 [26]. Subsequently, caspase-9 can lyse and activate effector caspases such as caspase-3 or caspase-7 [27,28]. Studies have reported that during viral infection, the ORF3 of PEDV inhibits the apoptosis of host cells, which is conducive to the replication and transmission of the virus [29]. Therefore, the induction of early apoptosis of PEDV-infected host cells and effective prevention of the replication and transmission of PEDV can be a novel strategy for PEDV treatment.

TCRV antiviral effects and mechanism of action were studied using Vero cells and IPEC-J2 cell models. TCRV promoted early apoptosis in PEDV-infected cells, and this pathway was activated by the mitochondria-related caspase pathway.

## 2. Results

### 2.1. TCRV Inhibits PEDV Infection In Vitro

**Figure 1:**
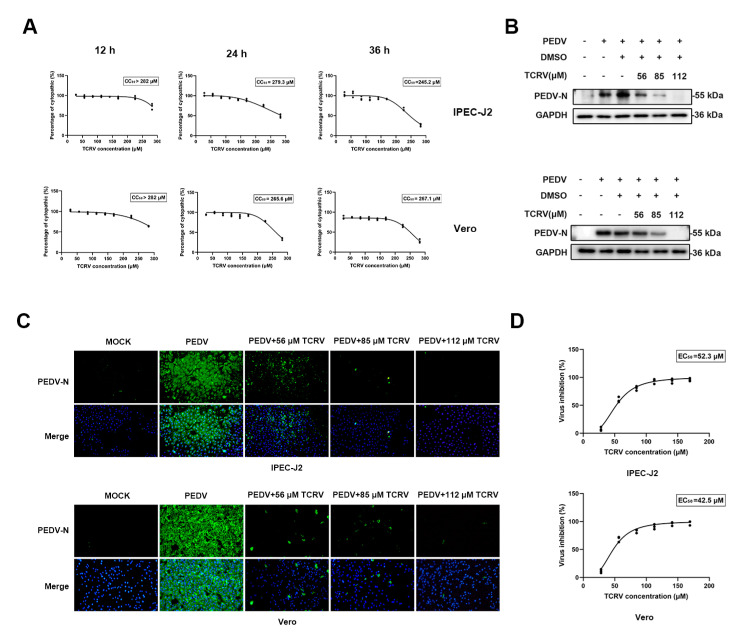
TCRV inhibits PEDV infection in Vero cells and IPEC cells. (**A**) Determination of the cytotoxicity of TCRV (28 µM, 56 µM, 85 µM, 112 µM, and 141 µM) by the CCK-8 assay. (**B**) Vero cells and IPEC cells were mock−infected or infected with PEDV (MOI = 0.5 and 2.5). The expressions of PEDV N protein were assessed using Western blotting after TCRV treatment (56 µM, 85 µM, and 112 µM). The results were presented as the ratio of the band intensities of the target protein to GAPDH. (**C**) The treatment of PEDV-infected cells with TCRV at concentrations of 56 µM, 85 µM, and 112 µM. Cells were processed for immunofluorescence using antibodies against PEDV N protein and DAPI staining. (**D**) Dose-response curve for PEDV N protein inhibition by TCRV using immunofluorescence assay.

Vero or IPEC-J2 cells were exposed to TCRV at diverse doses for 12, 24, and 36 h. CCK-8 assays indicated that TCRV at 56 µM, 85 µM, and 112 µM did not display cytotoxicity on uninfected cells (Figure 1A). To investigate TCRV’s impact on PEDV infection, we infected Vero or IPEC-J2 cells by PEDV CV777 (MOI = 0.5 and 2.5) [30] for 1 h and subsequently exposed them to 56 µM, 85 µM, and 112 µM TCRV for 24 h. The cells were gathered to assess PEDV N protein expression. As shown in the Western blotting assay, treatment with TCRV lowered PEDV N protein expression (Figure 1B). An indirect immunofluorescence assay indicated the decreased infected cell number of TCRV-treated group compared to the PEDV-infected group in a dose-dependent manner (Figure 1C). We determined the EC_50_ values (drug concentration required to reduce infection by 50%) of TCRV against PEDV infection in Vero and IPEC-J2 cells (Figure 1D). The selective index of TCRV (SI) was 5.34 and 6.25.

**Figure 2:**
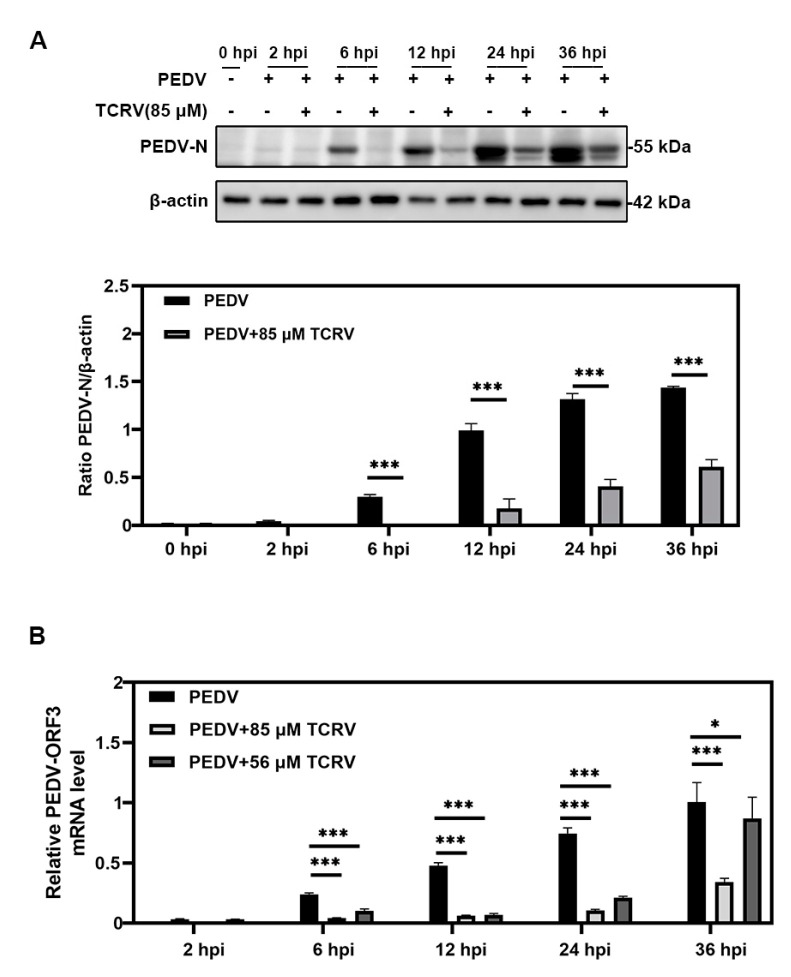
Inhibitory effect of TCRV on PEDV infection at different time points. (**A**) PEDV-infected cells treated with TCRV at different concentrations at 2, 6, 12, 24, and 36 hpi were analyzed by Western blotting. The PEDV N protein band was quantified as the ratio of the band intensities of PEDV N protein to β-actin in Western blotting. (**B**) PEDV-infected cells treated with TCRV at different concentrations at 2, 6, 12, 24, and 36 hpi were analyzed by RT-qPCR. Data from three independent experiments and error bars are presented as the mean ± SEM. * *p* < 0.05, *** *p* < 0.001.

To explore whether TCRV had a long-acting inhibitory effect on PEDV infection, we treated PEDV-infected cells with 85 µM TCRV for different periods at 2, 6, 12, 24, and 36 h post infection (hpi). The cells were then collected for the Western blotting assay. The Western blotting assay indicated the reduced PEDV N protein expression with time of exposure of PEDV-infected cells to TCRV (Figure 2A). Moreover, we conducted an RT-qPCR assay to examine PEDV ORF3 levels at different time points. It also demonstrated that the RNA of PEDV-ORF3 decreased significantly after the treatment of PEDV-infected cells with TCRV (Figure 2B). These data suggested that treatment with TCRV could inhibit PEDV infection in vitro within 36 hpi.

### 2.2. TCRV Promotes PEDV-Induced Apoptosis In Vitro

**Figure 3:**
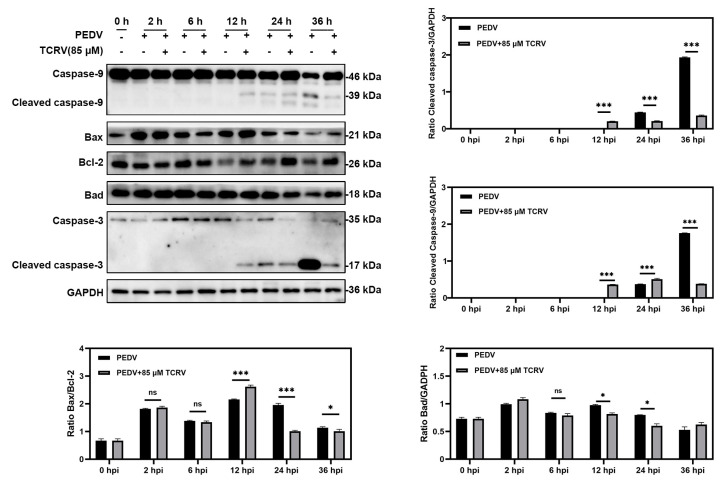
TCRV promotes PEDV-induced apoptosis in Vero cells. Western blotting analysis of activation caspase-3, –9, Bax, Bcl-2, and Bad proteins in PEDV-infected Vero cells treated with the indicated concentrations of TCRV at 24 hpi. Results are presented as the ratio of band intensities of the target protein to GAPDH. Data from three independent experiments and error bars are presented as the mean ± SEM. * *p* < 0.05, *** *p* < 0.001, ns, not significant.

**Figure 4:**
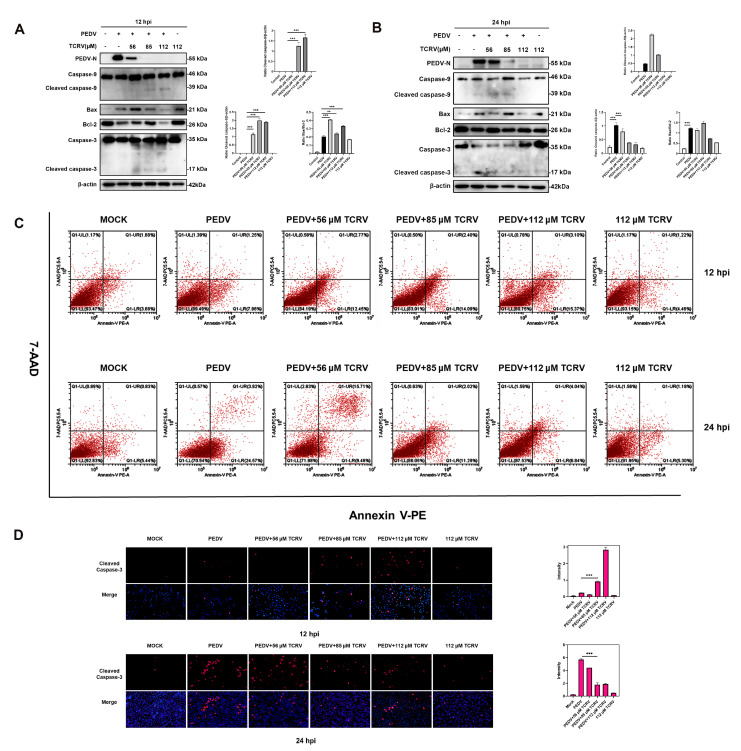
The apoptosis induced by TCRV in PEDV-infected cells is different from PEDV-induced apoptosis. (**A**, **B**) Western blotting analysis of PEDV N, caspase-3, -9, Bax, and Bcl-2 proteins in PEDV-infected Vero cells treated with the indicated concentrations of TCRV at 12 hpi and 24 hpi. Results were presented as the ratio of band intensities of the target protein to β-actin. (**C**) PEDV-infected cells were treated with the indicated concentrations of TCRV at 12 hpi and 24 hpi. Apoptosis was analyzed using Annexin V-PE/7AAD staining, and the apoptotic cells were explored using flow cytometry. The Annexin V-positive cells were considered apoptotic cells. (**D**) The treatment of PEDV-infected cells with TCRV at concentrations of 56 µM, 85 µM, and 112 µM. Cells were processed for immunofluorescence using antibodies against cleaved caspase-3 protein and DAPI staining. Data from three independent experiments and error bars are presented as the mean ± SEM. ** *p* < 0.01, *** *p* < 0.001.

Previous research demonstrated that PEDV could induce apoptosis in vitro [31]. Interestingly, apart from the PEDV group, we also found some dead cells in the group PEDV treated with TCRV. Early apoptosis is also an antiviral strategy to prevent the further spread of the virus. Then, we selected different time points to investigate the specific mechanism of TCRV antivirals. TCRV increased the Bax/Bcl-2 ratio and activated Caspase-3 and Caspase-9 rather than Bad in PEDV-infected cells at 12 hpi. (Figure 3). Two time points (12 hpi and 24 hpi) were chosen to further distinguish TCRV-induced apoptosis from PEDV-induced apoptosis. The Western blotting assay showed that TCRV activated the Caspase-3 and Casepase-9 protein before only PEDV in infected cells at 12 hpi. TCRV inhibited PEDV-induced apoptosis at 24 hpi. The results of the activated Caspase-3 indirect immunofluorescence assay revealed that, in comparison to the TCRV group, the PEDV group did not have many positive cells at 12 hpi (Figure 4D). Surprisingly, the number of positive cells in the PEDV group was greater than that in the TCRV group at 24 hpi. (Figure 4D). These findings suggest that TCRV may have induced apoptosis in PEDV-infected cells prior to PEDV-induced apoptosis. In addition, the Annexin-V/7-AAD staining was used to further validate our findings. The number of apoptotic cells in the PEDV-TCRV group was higher than in the PEDV group at 12 hpi. The apoptotic rate was reduced at 24 hpi in response to TCRV treatment (Figure 4C). TCRV promoted PEDV-induced apoptosis in vitro and cleared PEDV-infected cells, according to these findings.

### 2.3. TCRV-Induced Mitochondrial Membrane Potential (MMP) Decreased in PEDV-Infected Cells

**Figure 5:**
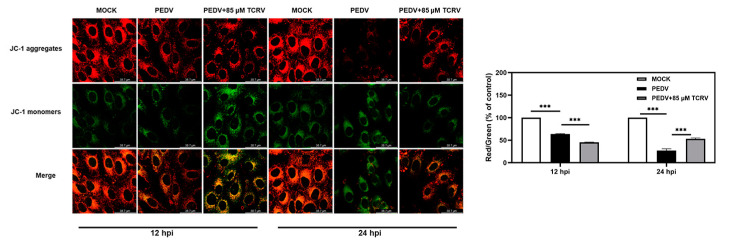
Effects of TCRV on mitochondrial membrane potential. (**A**) The mitochondrial membrane potential of PEDV-infected Vero cells treated with the indicated concentrations of TCRV at 12 hpi and 24 hpi. The fluorescence signals were visualized using confocal immunofluorescence microscopy. Results were presented as the ratio of JC-1 aggregate (red) to JC-1 monomer (green). Data from three independent experiments and error bars are presented as the mean ± SEM. *** *p* < 0.001.

The main indicator of mitochondrial injury is the decline in mitochondrial membrane potential (MMP). JC-1 is a fluorescence tracker for the detection of MMP, which can be transformed from the polymeric form (red fluorescence) to the monomeric form (green fluorescence) in conditions of low MMP. Therefore, the JC-1 assay was used to measure mitochondrial dysfunction in Vero cells at two time points (12 hpi and 24 hpi). We observed that the ratio of red to green fluorescence in the TCRV group was lower compared to the PEDV group at 12 hpi. At 24 hpi, the ratio of red to green fluorescence increased in the TCRV group (Figure 5A). These data demonstrated that TCRV-induced MMP was decreased in PEDV-infected cells in the early stage of PEDV infection. However, the mitochondrial membrane potential was increased after that.

### 2.4. TCRV-Induced Cytochrome C Translocation after PEDV Infection

**Figure 6:**
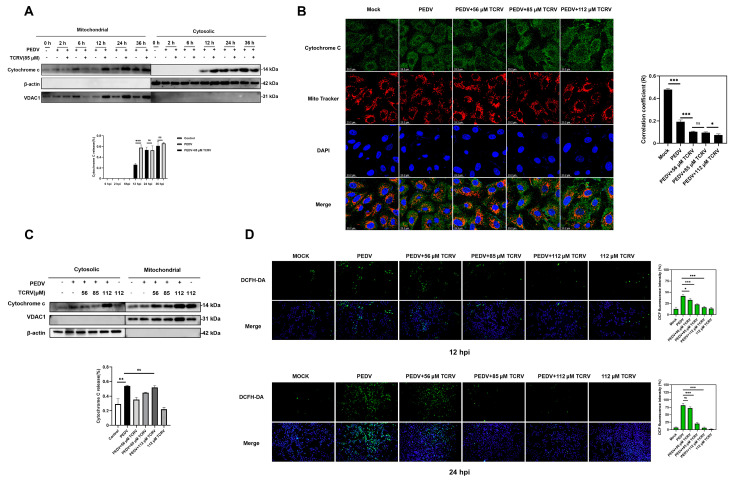
TCRV promotes the release of cytochrome C from the mitochondria. (**A**) To analyze the subcellular localization of cytochrome C, PEDV-infected cells treated with TCRV at different concentrations at 2, 6, 12, 24, and 36 hpi were fractionated and mitochondrial and cytosolic fractions were subjected to Western blotting. VDAC1 was used as mitochondrial marker and β-actin was used as cytosolic marker (**B**) The treatment of PEDV-infected cells with TCRV at concentrations of 56 µM, 85 µM, and 112 µM for 24 h. Images were acquired by confocal laser-scanning microscopy, detecting cytochrome C (green), mitochondria (red), and nuclei (blue), which were visualized by DAPI (scale bar, 23.2 µm). Images were collected on a Leica SP8 Laser Scanning confocal microscope. Pearson’s correlation coefficient analysis was carried out using Image J software. (**C**) The treatment of PEDV-infected cells with TCRV at concentrations of 56 µM, 85 µM, and 112 µM for 24 h were fractionated and mitochondrial and cytosolic fractions were subjected to Western blotting. VDAC1 was used as a mitochondrial marker and β-actin was used as a cytosolic marker. (**D**) Intracellular ROS levels were detected by DCF fluorescence intensity. The graph presents the production of ROS in the PEDV-infected cells treated by TCRV. Data from three independent experiments and error bars are presented as the mean ± SEM. * *p* < 0.05, ** *p* < 0.01, *** *p* < 0.001, ns, not significant.

The mitochondrial pathway, an intrinsic apoptosis activation pathway, is activated by stimuli that cause the outer mitochondrial membrane (OMM) to permeabilize and proteins to be released from the mitochondrial intermembranous space (IMS). One of these proteins is cytochrome C. (27). Mitochondria were isolated from Vero cells at various times after PEDV infection to assess the diffusion of cytochrome C from mitochondria. Cytochrome C was detected in the cytoplasm 12 h after PEDV infection, as shown by the Western blotting assay, but the release of cytochrome C in the TCRV treatment group was greater than that in the PEDV group. We discovered that there was no difference in cytochrome C release between the TCRV treatment group and the PEDV treatment group after 24 h (Figure 6A). The co-localization of cytochrome C with mitochondria in infected cells was further demonstrated by an immunofluorescent assay at 24 hpi (Figure 6B). Furthermore, TCRV-induced cytochrome C release was dose-dependent (Figure 6C). This release is not related to ROS production (Figure 6D). These findings showed that PEDV could induce cytochrome C release from mitochondria, but this process was accelerated after TCRV treatment.

### 2.5. TCRV Inhibits PEDV Infection by the Regulation of Caspase-3

**Figure 7:**
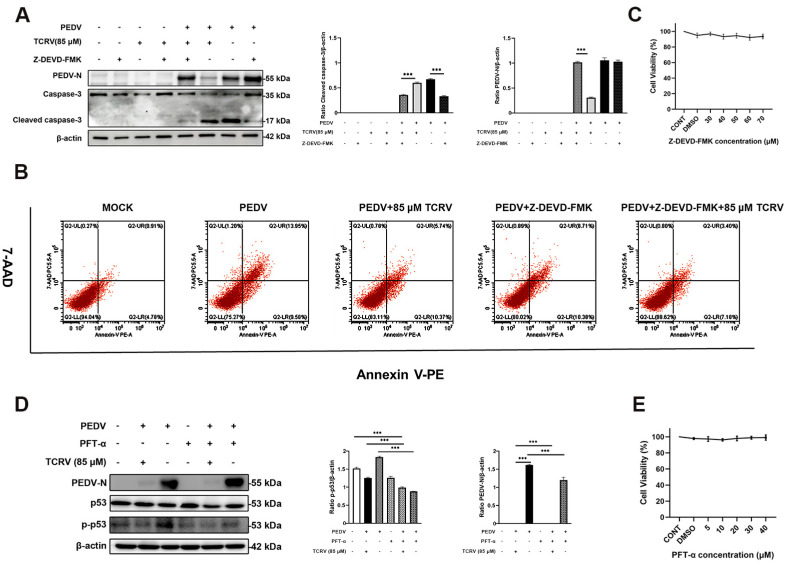
TCRV inhibits PEDV infection through the regulation of caspase-3. (**A**) Western blotting analysis of PEDV N, cleaved caspase-3, and caspase-3 proteins in PEDV-infected Vero cells treated with 85 µM TCRV and 50 µM Z-DEVD-FMK at 24 hpi. Results were presented as the ratio of band intensities of the target protein to β-actin. (**B**) PEDV-infected cells were treated with 85 µM TCRV and 50 µM Z-DEVD-FMK at 24 hpi. Apoptosis was analyzed using Annexin V-PE/7AAD staining, and the apoptotic cells were explored using flow cytometry. The Annexin V-positive cells were considered apoptotic cells. (**C**) Determination of the cytotoxicity of Z-DEVD-FMK (30 µM, 40 µM, 50 µM, 60 µM, and 70 µM) by the CCK-8 assay. (**D**) Western blotting analysis of PEDV N, p-p53, and p53 proteins in PEDV-infected Vero cells treated with the indicated concentrations of TCRV and PTF-α (20 µM) at 24 hpi. Results were presented as the ratio of band intensities of the target protein to β-actin. (**E**) Determination of the cytotoxicity of PTF-α (5 µM, 10 µM, 20 µM, 30 µM, and 40 µM) by the CCK-8 assay. Data from three independent experiments and error bars are presented as the mean ± SEM. *** *p* < 0.001.

We further aimed to understand the relationship between the inhibition of PEDV and the promotion of apoptosis of PEDV-infected cells by TCRV. We pretreated Vero cells with the Caspase-3 inhibitor Z-DEVD-FMK for 2 h and subsequently infected the cells with PEDV in the presence of varying concentrations of TCRV for 24 h. The results of western blotting demonstrated that the antiviral effect of TCRV was significantly reduced after the simultaneous treatment of PEDV-infected cells by Z-DEVD-FMK and TCRV (Figure 7A). Moreover, the apoptosis of PEDV-infected cells due to the impairment of cell viability by TCRV was further supported by flow cytometric analysis (Figure 7B). CCK-8 assays indicated that Z-DEVD-FMK at 50 µM did not display cytotoxicity in Vero cells (Figure 7C). These data indicated that TCRV inhibited PEDV infection through the regulation of caspase-3.

To investigate whether the protein p53 was involved in the suppression of PEDV infection by TCRV, we pretreated Vero cells with the p53-inhibitor PTF-α for 2 h and subsequently infected the cells with PEDV in the presence of varying concentrations of TCRV for 24 h. The results of the wWestern blot revealed that the antiviral effect of TCRV continued to exist (Figure 7D). CCK-8 assays indicated that PTF-α at 20 µM did not display cytotoxicity in Vero cells (Figure 7E). These data suggested that the antiviral effect of TCRV was independent of p53.

## 3. Discussion

Owing to the excellent and extensive antiviral pharmacological effects of resveratrol [32], we investigated its ability to inhibit PEDV. TCRV is a resveratrol derivative that has been proven to possess better pharmacological activity than resveratrol [33]. Previous studies have reported that TCRV showed a similar reactivation effect on latent HIV as RSV [34]. In this study, we analyzed its derivative TCRV and observed that it was highly effective in inhibiting PEDV.

After observing the excellent viral inhibitory effects of TCRV, we noticed that it could promote the early apoptosis of PEDV-infected cells. Apoptosis is a tightly controlled multistep process of cell death that occurs in response to an extensive range of stimuli, including viral infection and drug interventions [27]. Virus-induced apoptosis is often a strategy of viruses to spread infection [35]. However, the blockage of apoptosis can avoid premature death of infected cells, thus allowing high titers of viral replication or persistent infection [36]. Existing research suggests that the ORF3 of PEDV could inhibit the apoptosis of host cells from subverting the antiviral function of the host [29]. Induction of early apoptosis of PEDV-infected cells would result in the inhibition of PEDV replication. We demonstrated that PEDV enhanced the expression of the pro-apoptotic protein and lowered the level of anti-apoptotic protein. After treating PEDV-infected cells with different concentrations of TCRV, we observed that TCRV significantly induced the early apoptosis of PEDV-infected cells, and upregulated the ratio of Bax/Bcl-2 compared to the PEDV group. Therefore, our next step was to determine the mechanism of induction of apoptosis of PEDV-infected cells by TCRV and to explore the specific pathways.

PEDV could induce caspase-independent apoptosis by activating the mitochondrial apoptosis-inducing factor [31,37]. The mitochondria-mediated caspase activation pathway is a major apoptotic pathway that is featured by the reduction of MMP, as well as the subsequent release of cytochrome C into the cytoplasm to activate caspases [27]. Our experiment proved that PEDV could cause the release of cytochrome C from mitochondria and reduce MMP. It could also promote the expression of mitochondrial apoptosis-related proteins. Compared to the PEDV group, the amount of cytochrome C released from mitochondria to cytoplasm increased, and the membrane potential of mitochondria decreased with the addition of TCRV in a dose-dependent manner. Moreover, the mitochondrial apoptosis-related proteins caspase-9, cytochrome C, and apaf1 also increased with an increasing dose of TCRV. These results indicated that TCRV could induce PEDV-infected cell apoptosis through mitochondria. Based on this, we used different time points to differentiate between PEDV-induced apoptosis and TCRV-induced apoptosis. The results indicated that TCRV could inhibit PEDV infection by inducing early apoptosis before PEDV-induced apoptosis.

According to previous studies, resveratrol could trigger apoptosis by activating p53 and increasing the levels of ROS [38,39]. Simultaneously, PEDV could cause cell apoptosis through a p53/ROS-dependent pathway [22,40]. In this study, we proved that PEDV at MOI = 0.5 could increase the activation of p53, as well as the ROS produced in vitro. Thus, we investigated whether TCRV could induce the apoptosis of PEDV-infected cells by activating p53 and whether they were associated with ROS. ROS fluorescence results showed that TCRV reduced the ROS production of PEDV, whether it was for 12 or 24 hpi. The Western blot assay indicated that after treatment with the p53 inhibitor PFT-α, the antiviral effect of TCRV continued to exist, and ROS levels decreased, indicating that the antiviral effect of TCRV was independent of p53 and not related to ROS.

**Figure 8:**
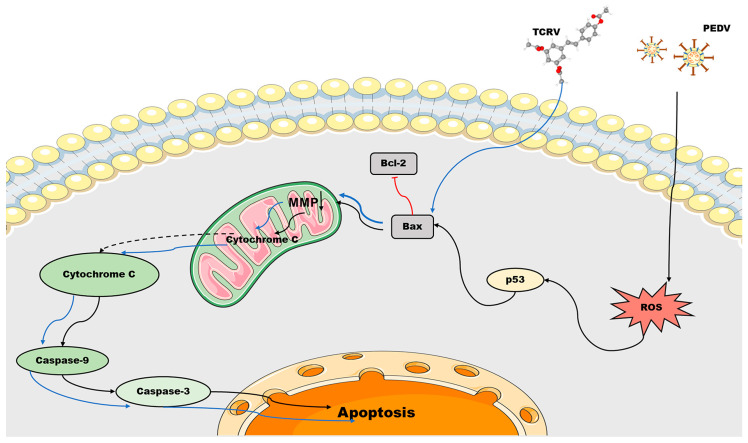
Model of apoptosis induced by the treatment of PEDV-infected Vero cells with TCRV. PEDV activates p53 to produce ROS and causes caspase-mediated mitochondrial apoptosis. TCRV directly mediates mitochondrial apoptosis induced by the caspase pathway, resulting in the early apoptosis of PEDV-infected cells and thus eliminating PEDV-infected cells.

To confirm that the antiviral effect of TCRV is closely related to apoptosis, we inhibited key apoptotic proteins. Treatment with TCRV significantly enhanced the expressions of the ratio of Bax/Bcl-2, activated caspase-3 and caspase-9, and stimulated the release of cytochrome C from mitochondria to the cytoplasm, indicating that the antiviral effect of TCRV was mediated by mitochondrial apoptosis. Therefore, we selected caspase-3 inhibitor Z-DEVD-FMK to confirm our findings. The results indicated that the antiviral effect of TCRV in significantly lowering the number of PEDV-infected cells was also notably reduced after treatment with Z-DEVD-FMK. Therefore, our results suggest that TCRV could directly mediate mitochondrial apoptosis induced by the caspase pathway, resulting in the early apoptosis of PEDV-infected cells and thus eliminating PEDV-infected cells. In conclusion, our results suggested that TCRV could induce the apoptosis of PEDV-infected cells through the activation of the mitochondria-related caspase pathway (Figure 8).

## 4. Materials and Methods

### 4.1. Cytotoxicity Assay

The cytotoxicity of TCRV (Chengdu Herbpurify Co., Ltd., Chengdu, China) PTF-α (Selleck, UT, USA), and Z-DEVD-FMK (Selleck, HOU, TX, USA) was evaluated in vitro by Cell Counting Kit-8 (CCK8, DOJINDO, Tokyo, Japan). We cultivated Vero cells (ATCC CCL-81) and IPEC-J2 cells with DMEM/F12 (Gibco, Grand Island, NY, USA) that contained 10% fetal bovine serum (FBS, Gibco, Grand Island, NY, USA) as well as 1% antibiotic-antimycotic (Gibco, Grand Island, NY, USA) at 37 °C with 5% CO_2_, which were grown to 70% confluence for 24 h. TCRV, PTF-α, and Z-DEVD-FMK were supplemented to the Vero cell culture, followed by 36-h incubation. Thereafter, we exchanged the culture medium with DMEM/F12 (100 µL) and CCK-8 solution (10 µL), followed by 2 h incubation under 37 °C. Later, we utilized the microplate reader (Infinite M200 PRO, TECAN, Hombrechtikon, Switzerland) for measuring the absorbance at 450 nm.

### 4.2. Cells and Virus

Vero cells, IPEC-J2 cells, and PEDV CV777 strain (GenBank accession no: AF353511) were used in this study. Vero cells were incubated to 70% confluence. PEDV was serially diluted (10-fold) within DMEM/F12, followed by the addition into Vero cells in 10 replicates per dilution. The viral titers were denoted to be TCID_50_, measured by the Reed and Muench approach [30].

### 4.3. Medicine Treatment

PEDV infection was performed as described in Section 4.2. PEDV (MOI = 0.5) was cultured in 2% FBS DMEM/F12 medium for 1 h and then rinsed thrice using PBS. Then, the culture was treated with 56 µM, 85 µM, and 112 µM of TCRV.

### 4.4. Indirect Immunofluorescence Assay (IFA)

The treated cells were subject to 15 min 4% paraformaldehyde (PFA, Solarbio, Beijing, China) fixation, followed by 10 min permeabilization using 0.2% Triton X-100 (Solarbio, Beijing, China) on ice. Later, we stained cells using an anti-PEDV monoclonal antibody (1:200, Medgene Lab, USA) or Cleaved-caspase-3 (1:200, Cell Signaling Technology, LA, CA, USA) for an 8 h period under 4 °C. After rinsing thrice, FITC-labeled goat anti-mouse IgG (1:800, Abcam, NY, USA) was used to incubate cells for 1 h. We further incubated cells using 4’,6-Diamidino-2’-phenylindole (DAPI, Solarbio, Beijing, China) for a 5 min period and subsequently analyzed them using fluorescence microscopy (Olympus CKX53, Tokyo, Japan).

### 4.5. Western Blotting (WB) Assay

All primary antibodies utilized in this study included Bax (1:1000), Bcl2 (1:1000), Bad (1:1000), Caspase-3 (1:1000), Cyt C (1:1500), Cleaved-Caspase9 (1:1500), Cleaved-caspase-3 (1:1000, all from Cell Signaling Technology, USA), p-p53 (1:1000), p53 (1:1000, Abcam, UK), PEDV N (1:1000), GAPDH (1:5000), β-actin (1:5000), and VDAC-1 (1:3000, ProteinTech Group, Wuhan, China). Secondary antibodies were peroxidase-conjugated secondary antibodies (1:1500, ProteinTech Group, Wuhan, China).

### 4.6. RNA Extraction and Quantitative RT-PCR(RT-qPCR)

We isolated total RNA in Vero cells by RNAiso Plus kit (Takara, Kyoto, Japan). Later, cDNA was prepared from total RNA by the PrimeScript RT reagent Kit based on a gDNA Eraser (Takara, Kyoto, Japan). RT-qPCR was conducted in thermal cycler ABI 7500 (Applied Biosystems, Foster City, CA, USA) for determining viral loads. The primers (Sangon, Shanghai, China) used in this study are presented in Table 1. In the gene expression analysis, the CT values of the PEDV-N genes were normalized to GAPDH and compared to the mock-infected control. The assay was conducted thrice. Data were measured as the fold-change based on the 2^−ΔΔCT^ method.

### 4.7. Annexin V-PE and 7-Amino-Actinomycin (7-AAD) Double-Staining Assay

Annexin V-PE and 7-Amino-Actinomycin double-staining kit (Vazyme, Nanjing, China) was used in this study. After collecting the cells, they were rinsed thrice using PBS and then centrifuged at 800 g. After removing supernatants, we resuspended cells in a 200 µL binding buffer in a flow tube, and Annexin-PE as well as 7-AAD (5 µL each) was added to the tube to incubate cells for a 15 min period in the dark and to be counted using flow cytometry (BD FACSCalibur, San Diego, CA, USA).

### 4.8. Determination of Mitochondrial Membrane Potential (MMP)

An MMP assay was performed according to the protocol described in the cellular experiment. The MMP of podocytes was determined using 5 µM JC-1 (Thermo, Grand Island, NY, USA). The cells were grown with JC-1 in the dark under 37 °C for a 30 min period and then rinsed by JC-1-washing buffer. Then, the fluorescence was determined using the Leica SP8 Laser Scanning confocal microscope (Leica Microsystems, Wetzlar, Germany).

### 4.9. Determination of the Levels of Intracellular Reactive Oxygen Species (ROS)

ROS was determined using the DCFH-DA Reactive Oxygen Species Assay Kit (DOJINDO, Tokyo, Japan). Vero cells were rinsed thrice using PBS and stained using 50 µM DCFH-DA (dissolved in DMEM/F12) for 30 min. ROS was detected at the FITC channel. The positive-staining cells were analyzed using fluorescence microscopy (Olympus CKX53, Tokyo, Japan).

### 4.10. Mitochondria Isolation and Cytochrome C Release Assays

Cells were harvested, and the Cell Mitochondria Isolation Kit (Beyotime, Shanghai, China) was used for mitochondria isolation. Mitochondrial samples were resuspended in mitochondrial lysate (Beyotime, Shanghai, China) supplemented with Phenylmethylsulfonyl fluoride (PMSF). The mitochondrial resuspension and cytoplasmic samples were analyzed by Western blotting. Cytochrome C release was analyzed by comparing band intensities of cytochrome C in mitochondria and cytoplasms.

### 4.11. Indirect Immunofluorescence Assay (IFA) to Analyze Cytochrome C Subcellular Localization

MitoTracker Red CMXRos (Solarbio, Beijing, China) was used for the cytochrome C assay. Treated cells were stained with 200 nM MitoTracker Red CMXRos for 45 min. Following fixation with 4% paraformaldehyde, we stained cells using anti-cytochrome C antibody (1:300, Cell Signaling Technology, USA) at 4 °C for 8 h, and incubated them using FITC-labeled goat anti-mouse IgG for 1 h and DAPI (Solarbio, Beijing, China). The Leica SP8 Laser Scanning confocal microscope (Leica Microsystems, Germany) was employed for fluorescence visualization and photographing.

### 4.12. Statistical Analysis

In this study, all the experiments were carried out in triplicate. The Student’s *t* test was performed to compare differences between means. Data were examined by software GraphPad Prism version 8 (GraphPad Software, San Diego, CA, USA) and presented as mean ± SEM. P-values of <0.05 stood for statistical significance.

## 5. Conclusions

TCRV effectively inhibited PEDV by activating the mitochondria-related apoptosis caspase pathway and clearing the PEDV-infected cells. Our research provides a novel strategy against PEDV.

## Figures and Tables

**Table 1:** Primers utilized in RT-qPCR.

Genes	Forward 5′-3′	Reverse 5′-3′
PEDV ORF3	TAGACAAGCTTCAAATGTGAC	GTATTAAAGATAATAAGGAGCGC
GAPDH	AGGTCGGAGTCAACGGATTT	TAGTTGAGGTCAATGAAGGG

## Data Availability

Data are contained within the article. The data presented in this study are available on request from the corresponding author.
